# DiPPI: A Curated Data Set for Drug-like Molecules in Protein–Protein Interfaces

**DOI:** 10.1021/acs.jcim.3c01905

**Published:** 2024-06-22

**Authors:** Fatma Cankara, Simge Senyuz, Ahenk Zeynep Sayin, Attila Gursoy, Ozlem Keskin

**Affiliations:** † Graduate School of Sciences and Engineering, Koç University, İstanbul 34450, Turkey; ‡ Department of Chemical and Biological Engineering, Koç University, İstanbul 34450, Turkey; § Department of Computer Engineering, Koç University, İstanbul 34450, Turkey

## Abstract

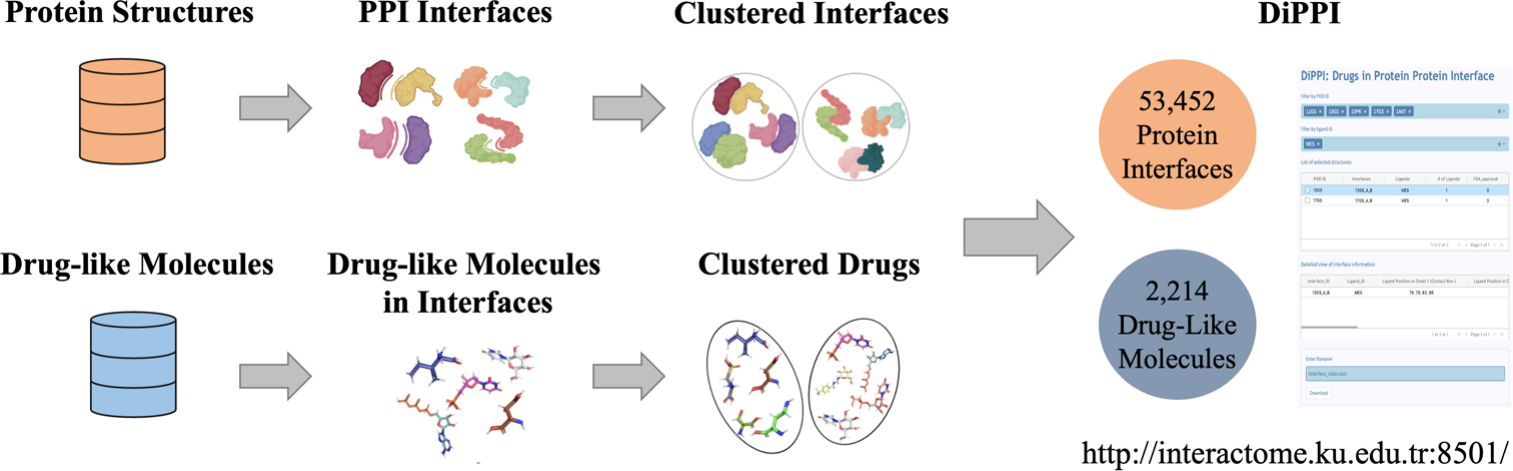

Proteins interact through their interfaces, and dysfunction of protein–protein interactions (PPIs) has been associated with various diseases. Therefore, investigating the properties of the drug-modulated PPIs and interface-targeting drugs is critical. Here, we present a curated large data set for drug-like molecules in protein interfaces. We further introduce DiPPI (Drugs in Protein–Protein Interfaces), a two-module web site to facilitate the search for such molecules and their properties by exploiting our data set in drug repurposing studies. In the interface module of the web site, we present several properties, of interfaces, such as amino acid properties, hotspots, evolutionary conservation of drug-binding amino acids, and post-translational modifications of these residues. On the drug-like molecule side, we list drug-like small molecules and FDA-approved drugs from various databases and highlight those that bind to the interfaces. We further clustered the drugs based on their molecular fingerprints to confine the search for an alternative drug to a smaller space. Drug properties, including Lipinski’s rules and various molecular descriptors, are also calculated and made available on the web site to guide the selection of drug molecules. Our data set contains 534,203 interfaces for 98,632 protein structures, of which 55,135 are detected to bind to a drug-like molecule. 2214 drug-like molecules are deposited on our web site, among which 335 are FDA-approved. DiPPI provides users with an easy-to-follow scheme for drug repurposing studies through its well-curated and clustered interface and drug data and is freely available at http://interactome.ku.edu.tr:8501.

## 1 Introduction

Drug repurposing, a convenient strategy for extending the utility of existing pharmaceuticals beyond their original indications, presents a more time- and cost-efficient strategy than conventional drug discovery methods.^1,2^ However, identifying new targets for repurposed drugs is challenging due to the tendency of drugs to interact with multiple targets that share similar binding cavities.^3−6^ Despite a lack of global similarity, proteins may share comparable local binding sites, particularly at their interfaces.^7−9^ Targeting protein interfaces with properties similar to the original drug target has emerged as a promising approach in drug repurposing, given the crucial role these interfaces play in mediating drug effects.^10^ Although therapeutically targeting protein–protein interactions presents difficulties, new discoveries about binding regions and critical contacts—such as hotspots—have sparked a renewed interest in exploring these potential therapeutic targets.^3,11,12^ Therefore, investigating the properties of these interactions is critical for the identification of drug targets.^13^

The strategic emphasis on structural clustering of interfaces, complemented by a physicochemical analysis of such sites, facilitates the identification of proteins sharing similar topologies. Moreover, the combination of ligand clustering with the structural organization of interfaces enables a more refined investigation of potential drug candidates within specific clusters. Similar to the search for interface properties, drugs sharing analogous physicochemical and structural characteristics aid in the finding of potential candidates for drug repurposing. This integrated approach is a discerning framework for identifying proteins that may act as off-target or novel targets in drug repurposing.

A few public databases that contain chemical and target information linked to PPIs^14−17^ have been made available thus far. However, despite the significance of ligand clustering and structural insights into interfaces, the need for comprehensive resources to provide detailed information on drug-binding interfaces remains a significant obstacle. A more exhaustive and accessible repository of such interface data would significantly augment the precision and scope of strategies to repurpose existing drugs, ultimately contributing to the acceleration and optimization of drug discovery efforts.

Here, we present a curated large data set for drug-like molecules in protein interfaces alongside the publicly available DiPPI (Drugs in Protein–Protein Interfaces) web site, where users can access protein interfaces extracted from PDB and their associated drug-like molecules and FDA-approved drugs. Additionally, several physicochemical properties for the interfaces and drugs are presented on the web site to guide users on the molecule and target selection. We further investigated the relevance of our results using a docking procedure to find alternative drug–target pairs in a case study. Our work presents extensive protein interface data by analyzing 534,203 computationally derived interfaces and up-to-date drug-like molecules that are associated with these interfaces. DiPPI is freely available for data access and downloads at http://interactome.ku.edu.tr:8501. Users can download their query results and batch data files for interface and drug-like molecule associations.

## 2 Materials and Methods

**Figure 1:**
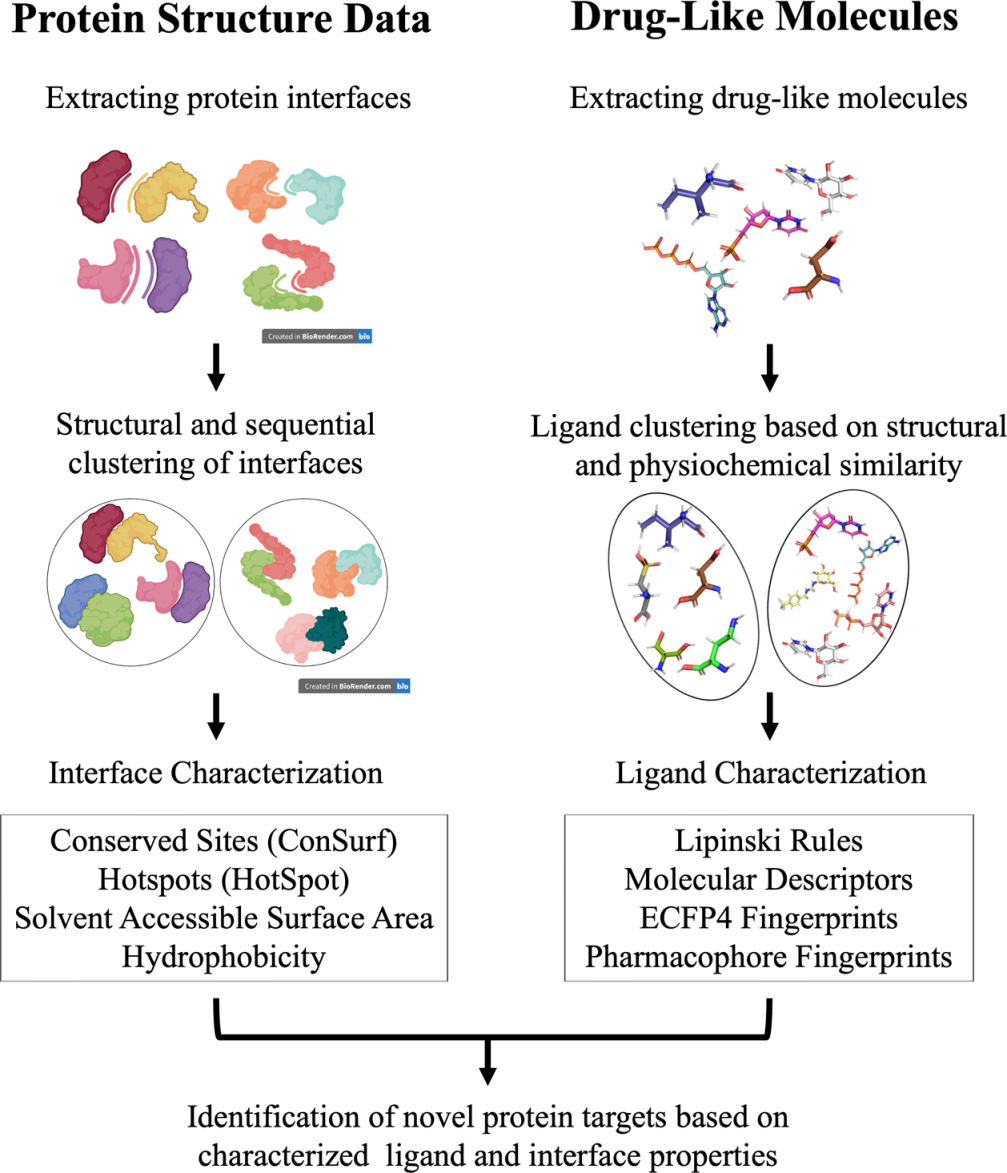
Flowchart of the DiPPI process. Interface clusters are created as described in Abali et al.^18^ Drug-like small molecule data are curated from five databases. Relevant physicochemical properties are calculated for interface and drug-like molecules.

DiPPI has two sites for protein interfaces and drug-like molecules (Figure 1). Merged data sets facilitate the identification of drug-like molecules targeting interfaces specifically.

### 2.1 Creating Interface Clusters

Interface clusters are created as described in Abali et al., 2021^18^ (see Supporting Information) as follows. First, all available PDB structures were downloaded, and possible dimers were identified based on their solvent available surface area (SASA). Further, distance criteria are used to define interfaces, as explained in the Supporting Information. Then, the interfaces were extracted from these dimers by identifying contacting and nearby residues. Contacting residues are described as those having heavy atoms from each of the chains separated by a distance smaller than the sum of their van der Waals radii and 0.5 Å. Nearby residues refer to the neighbors of interface residues in contact. In order to enhance interface continuity and preserve secondary structures, we extend the interface definition to include these neighboring residues along with the directly contacting interface residues. In addition, these residues are important in structural comparisons of interface regions. Any residue with a Cα atom within 6 Å of a contacting residue atom is defined as a nearby residue. The data set comprises 534,203 interfaces from all available 3D structures in PDB as of March 2022, representing different chains of 98,632 PDB structures. Interfaces are clustered by structural similarity using agglomerative hierarchical clustering as in our previous works.^18^ Representatives are selected as interfaces that are the most similar to all other interfaces in the same cluster.

### 2.2 Identification and Characterization of Small Drug-like Molecules

One of the critical steps in drug repurposing studies is the selection of drugs or small molecules to be repurposed. In this study, seven different databases, namely the LigandExpo database of PDB (http://ligand-expo.rcsb.org),^19,20^ ChEMBL,^21^ iPPIdb,^16^ PDID,^22^ Open Targets Platform,^23^ BindingDB,^24^ and ZINC database,^25^ were used to acquire drug-like molecule data. Molecules are labeled as drug or drug-like based on their definitions in the source databases. First, the small molecules deposited to the PDB are collected from LigandExpo. However, since not all of the molecules here have drug-like properties, molecules specified as drugs or drug-like molecules in the ChEMBL, iPPIdb, PDID, Open Targets Platform, and BindingDB databases were selected and included in the research. The PDID database provides access to a comprehensive set of putative and native protein–drug interactions in the human proteome. Drugs in the Open Targets Platform are defined as any bioactive molecule with drug-like properties as described in the EMBL-EBI ChEMBL database. BindingDB contains compounds from the FDA database for which BindingDB has binding affinity data; therefore, its molecules are also drugs. Small molecules from ChEMBL are retrieved from the drug section of the database. In the case of iPPIdb, the molecules are PPI modulators. Although they are not drugs, they have the capability to interfere with protein interface. Molecules from each data set are merged in a single set by removing duplicates and selecting only those having an entry in LigandExpo. This data set is further curated to eliminate cofactor, coenzyme, and ion molecules as annotated in PDBe.^26^ The resulting data set is hereby referred to as the drug-like data set (Figure S1). The count distribution of the drug-like molecules in the previously mentioned databases is given in Figure S2. After the drug-like molecule collection is used, a subset of this data set is generated based on whether these drugs are FDA-approved or not. Their FDA-approval status is taken from the ZINC database.^25^

### 2.3 Identification of Drug-Like Molecules in the Interfaces

After compiling drug-like molecules, we filtered molecules interacting with proteins constituting an interface in our interface data set. To further identify small molecules that are specifically in interfaces, we conducted a residue mapping using PDBsum^27^ (http://www.ebi.ac.uk/pdbsum). If the drug-associated residues were found within the interfaces, we labeled them as small-molecule-binding interface residues. Our data set comprises drug-like molecules that bind to both contacting residues and nearby residues, as we define the interfaces to include both of these residue types.

### 2.4 Data Set Characterization

For the characterization of drug-like molecules and FDA-approved drugs, various molecular descriptors are calculated with the Python RDKit module (2021_03_5), including but not limited to lipophilicity (logP), molecular weight, polar surface area (PSA), the number of rings, the number of carbons, the number of heteroatoms, the number of rotatable bonds (ROTB), the number of hydrogen bond acceptors (HBA), number of hydrogen bond donors (HBD), octanol–water partition coefficient (ALOGP), BalabanJ value, and BertzCT indices. The agreement to drug-likeness criteria of Lipinski,^28^ Ghose,^29^ Veber,^30^ Egan,^31^ and Muegge,^32^ as well as their quantitative estimate of drug-likeness (QED)^33^ was evaluated. Details of the selected criteria are given in Supporting Information.

For the characterization of interfaces, the following properties are included: basic characteristics (i.e., hydrophilicity, hydrophobicity, and polarity) of interface residues are calculated using an in-house script, hotspots are retrieved using HotPoint,^34^ conservation scores are computed from ConSurf-DB,^35^ and ligand binding energy values are collected from BindingDB.^24^

### 2.5 Small Molecule Clustering

We clustered small molecules to exploit their similarities in searching for an alternative small molecule. Structurally and physicochemically similar molecules are assumed to carry similar functions and be involved in similar processes.^36,37^ Using molecular fingerprint analysis, we clustered drug-like small molecules according to their structural and pharmacological properties. In this direction, we extracted extended connectivity fingerprints (ECFP4) and pharmacophore fingerprints of small molecules using SMILES profiles and calculated fingerprint similarity using the Tanimoto coefficient. Each fingerprint type offers unique ways to represent molecules and aids in assessing ligand similarity and clustering based on shared properties. ECFP highlights local structural patterns, while pharmacophore fingerprints identify similar pharmacological profiles.^38−41^ Integrating both provides a thorough analysis of potential drug repurposing opportunities, considering both structural and functional aspects.

After extracting fingerprints and quantifying their similarity, they are clustered using the Butina algorithm,^42^ a preferred clustering method in the pharmacological context.^43−45^ We evaluated distances between molecules by computing the 1-Tanimoto coefficient. After carefully examining the clustering results, we determined the optimal threshold values to be 0.6 for ECFP4 and 0.5 for pharmacophore fingerprints, as shown in Table S1. The clustering results depend on the distance cutoff, with higher thresholds yielding fewer clusters containing more similar molecules. Conversely, lower thresholds generate smaller clusters and individual “singletons”. Using these thresholds, the ECFP4 fingerprint similarity between two random molecules within a cluster is found to be 0.66, while the similarity between two random molecules in two random clusters is 0.07. These numbers are 0.65 and 0.16 for pharmacophore fingerprints, respectively. Drug-like molecules appearing in the same cluster are provided on the web site for each ligand.

### 2.6 Docking for Case Study

In this study, we performed docking using AutoDock Vina,^46^ and MGLTools^47^ was used for the receptor and ligand preparation. The 3-dimensional structures of drugs at reference pH are downloaded from the ZINC database.^25^ Additionally, BioPython^48^ and NumPy^49^ packages in Python and Open Babel^50^ are used. Protein-disease relation is found from DisGeNET.^51^

## 3 Results and Discussion

### 3.1 Drug-like Molecules

**Figure 2:**
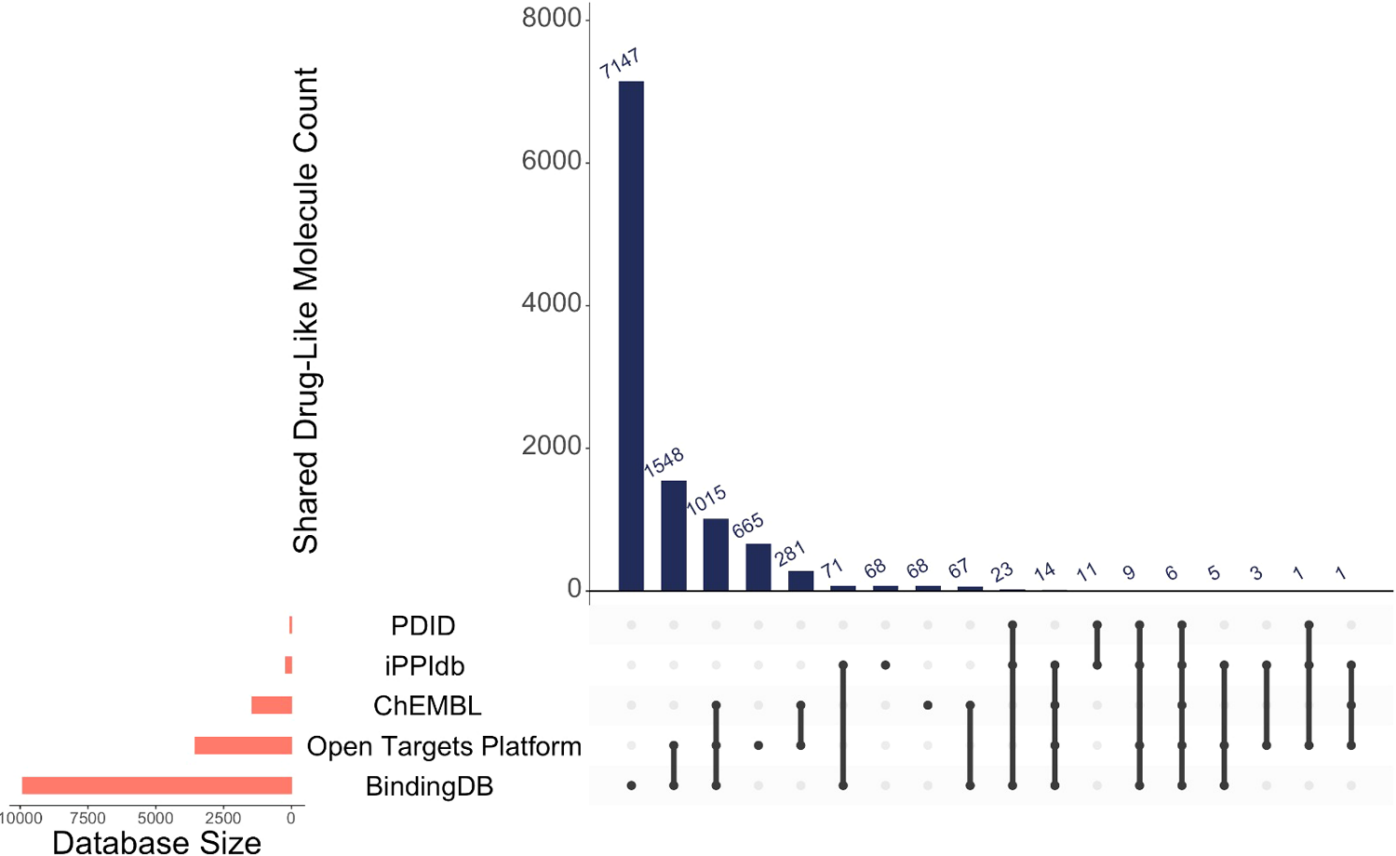
Distribution of drug-like small molecules with respect to the source database. Single dots show the individual contribution from each source, while connections show the mutual contribution of connected sources. The numbers on top show the total number contributed by each entry. For example, 7147 molecules are solely found in BindingDB, whereas 1548 molecules exist in the Open Targets Platform and BindingDB. Database size represents the total number of drug-like molecules each data source contributes.

Filtered data from selected databases contains 11,011 nonredundant small molecules known to have potential drug-like properties (see Section 2.2) and possess structure information in PDB (Figure 2). 2,214 of 11,011 molecules are found to be bound to at least one interface in our data set of 335,648 interfaces. A list of selected drug-like small molecules and eliminated molecules can be found on the web site.

We also retrieved FDA-approved drugs from the ZINC database.^25^ 620 out of 1615 nonredundant FDA-approved drugs bind to a protein structure (anywhere in the protein). 335 of the 620 drugs bind to the interfaces, and 242 of 335 bind to contacting residues. The list of these drugs can be found on the DiPPI GitHub repository. It can also be accessed through the DiPPI web site.

### 3.2 Protein–Protein Interfaces

Out of the initial 335,648 interfaces, only 63,882 contained a small molecule bound to the interface region. Among these, drug-like molecules bind exclusively to contacting residues in 4,096 cases and to nearby residues in 20,373 interfaces. On average, each drug-like molecule binding interface has 64.0 contacting residues and 77.3 nearby residues considering both sides of the interfaces. Contacting residues make up approximately 43.5% of the residues in these interfaces, while nearby residues comprise 56.4%.

Interfaces are further filtered based on the FDA approval of the bound drug. As a result, 19,960 interfaces were found to have at least one FDA-approved drug associated with them. The resultant data set is described in Table S2. Among these interfaces, 1,465 of them have an FDA-approved drug bound to only the contacting residues. FDA-approved drugs are only bound to the nearby residues in 5862 of the interfaces. The remaining 12,633 interfaces have an FDA-approved drug bound to both contacting and nearby residues.

The amino acid composition of each drug-like molecule-bound interface is analyzed for hydrophobic, polar, positively charged, and negatively charged residues. Interfaces predominantly feature hydrophobic residues, consistent with their buried locations between proteins. Both drug-like molecule-bound and FDA-approved drug-bound interfaces exhibit similar physicochemical properties. For drug-like molecule-bound interfaces, hydrophobic residues account for a mean percentage of 43.84% compared to 43.63% for FDA-approved drug-bound interfaces. Positively charged residues constitute 14.84% of drug-like molecule-bound interfaces and 14.34% of FDA-approved drug-bound interfaces. Negatively charged residues make up 10.41% of drug-like molecule-bound interfaces and 10.26% of FDA-approved drug-bound interfaces.

19,960 FDA-approved drug-bound interfaces cover a diverse range of proteins, indicating a comprehensive representation of different biological functions and processes. 11,113 unique proteins corresponding to 2378 Pfam families and 423 KEGG pathways are represented. The details for the ten most occurring KEGG pathways and Pfam families are given in Tables S3 and S4, respectively. Not surprisingly, overview pathways such as metabolic pathways (KEGG id: 01100), which are a combination of multiple pathways, are the most populated in our data set (Table S3). Many drugs target enzymes in various metabolic pathways to inhibit or enhance their activity.^52^ In addition, some disease-related pathways are also highly populated, such as the Alzheimer’s disease pathway (KEGG id: 05010) and the nonalcoholic fatty liver disease pathway (KEGG id: 04932). Given that the data set contains FDA-approved drug-bound interfaces, proteins implicated in disease-related pathways may make suitable targets for future drug repurposing. Some of the most populated Pfam families in our data set are the immunoglobulin C1-set domain (Pfam id: PF07654) and neurotransmitter-gated ion-channel ligand binding domain (Pfam id: PF02931) Table S4. Immunoglobulin C1-set domain is a classical antibody constant domain-like region commonly found in various immune system proteins. Immunoglobulin-related proteins such as Fc receptors are promising targets for various diseases, including inflammation and infectious diseases.^53^ In addition, ligand-gated ion channel proteins play a significant role in drug discovery. Several drugs target interfaces between subunits of the ion gates since the interfaces of these subunits have an important role in rearranging during gating.^54^

### 3.3 Ligand Clustering

**Figure 3:**
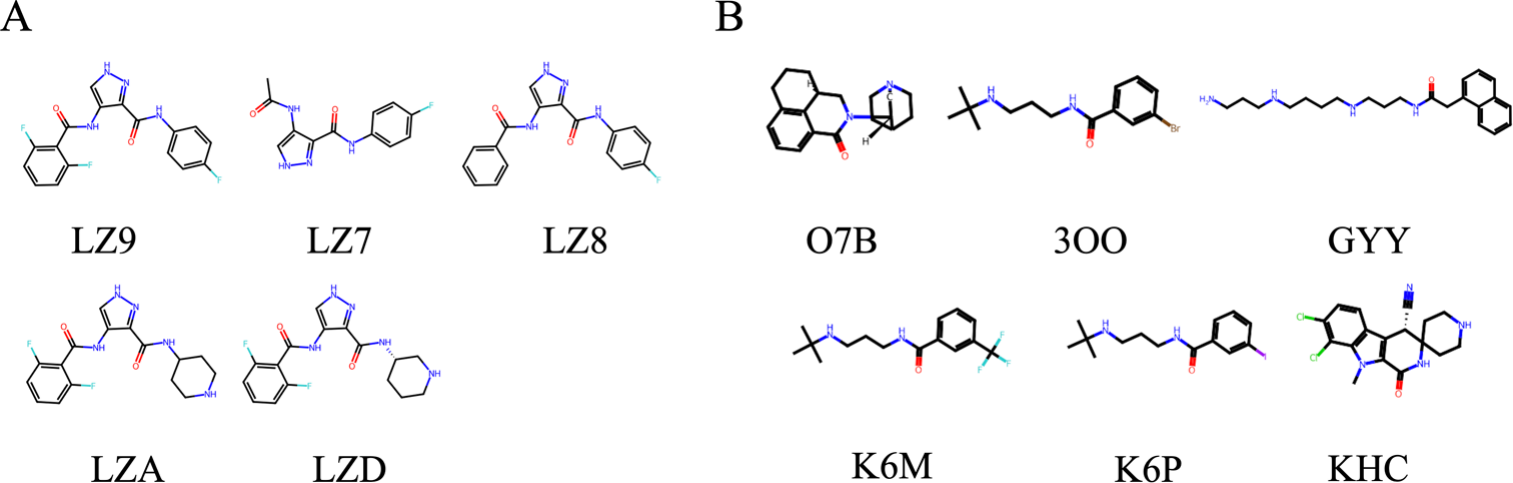
Members of a randomly selected cluster for (a) ECFP4 fingerprints and (b) pharmacophore fingerprints.

Members from an example cluster of ECFP4 and pharmacophore fingerprints can be seen in Figure 3. Molecules in the ECFP cluster share at least three aromatic rings connected with linear branches, except for LZ7, which has 2. This shared topology gives them a similar structure, which can be detected using ECFP fingerprints. On the other hand, in the pharmacophore cluster, six molecules are presented with possible similar pharmacological action. Among them, O7B is an approved drug (DrugBank ID: DB00377), which is an antagonist of 5-HT3 receptors that is indicated for the prevention and treatment of chemotherapy-induced nausea and vomiting.^55^ Although others do not have a discovered pharmacological activity, being in the same cluster may highlight them as candidates for investigation. Molecules in the same cluster from both fingerprint analyses are given in the data table provided on the web site. Users can process their results for either method upon download. As ECFP4 and pharmacophore fingerprints encode different characteristics regarding the ligand, using ECFP4 and pharmacophore fingerprints together will provide better insight into their characteristics.

### 3.4 Properties of Drugs Bound to Interfaces

Molecular descriptors of all drug-like molecules at interfaces are calculated and filtered based on the criteria from Lipinski, Ghose, Veber, Egan, Muegge, and QED. Of 11,011 interface-bound drug-like molecules, 7905 of them have no violations of Lipinski’s rules, 4799 of them have no violations of the Ghose criteria, 9595 of them have no violations of the Veber criteria, 9011 of them have no violations of the Egan criteria, and 4650 of them have no violations of the Muegge criteria. Of 335 FDA-approved drugs that are bound to at least one interface, 228 of them have no violations of Lipinski’s rules, 103 of them have no violations of the Ghose criteria, 275 of them have no violations of the Veber criteria, 253 of them have no violations of the Egan criteria, and 123 of them have no violations of the Muegge criteria.

The distribution of some of the molecular descriptors and the QED scores for FDA-approved and not-approved ones are given in Figure S3. FDA-approved ones seem to have less molecular weight and smaller log *P*, less molar refractive values, and a higher number of H-bond acceptors. The traditional range of molecular weight is between 200–500 Da.^28^ Based on the distribution of molecular weights in our data, we observed that 62.1% of FDA-approved drugs and 71.7% of non-FDA-approved drug-like molecules fall within this range, showing adherence to Lipinski’s Rule of Five. FDA-approved drugs tend to be lighter, with 22.7% weighing below 200 Da, whereas 13.2% of drug-like molecules not approved by the FDA fall within this weight range. Most drugs in our data set have 4–10 hydrogen acceptors, as per Lipinski’s rules and PPI inhibitors criteria.^28,53^ 51.8% of FDA-approved drugs and 67.4% of nonapproved drugs fall in this range. The QED score, indicating drug-likeness, ranges from 0 to 1, where 1 is the most likely to have drug-like properties. Bickerton et al. calculated 0.67 as the average QED score of drug-like molecules, which can be considered a drug-likeness threshold. In our data set, 25.7% of FDA-approved drugs and 24.9% of other drug-like molecules exceed this threshold. Those least likely to be drugs have an average QED score of 0.34. 29.3% of FDA-approved drugs and 26.1% of drug-like small molecules fall below the QED score of 0.4.

When the properties of drugs within and outside interfaces are examined using selected descriptors, notable differences in distributions emerge, as given in Figure S4. For instance, drugs within interfaces tend to have lower weights than those outside. Although PPI inhibitors can have molecular weights exceeding 500 Da, most FDA-approved drugs, whether within or outside interfaces, typically fall within the 200–500 Da range, aligning with Lipinski’s rules. Approximately 27.2% of FDA-approved drugs binding to interfaces and 25.8% of those not in the interfaces exceed this weight range. 18.5% of interface-bound and 11.0% of noninterface FDA-approved drugs are heavier than 500 Da in our data set, consistent with existing literature. The distribution of hydrogen bond acceptors among drug-like molecules differs between interface-bound and noninterface-bound drugs. Specifically, 52.6% of interface-bound FDA-approved drugs have 4–10 hydrogen acceptors, while 61.2% of noninterface-bound FDA-approved drugs fall within this range. Furthermore, 30.1% of interface-bound and 37.3% of noninterface-bound FDA-approved drugs have a QED score above 0.67, indicating high drug-likeness. Conversely, 29.2% of interface-bound and 15.3% of noninterface-bound FDA-approved drugs have a QED score below 0.4.

### 3.5 Web Site Overview

**Figure 4:**
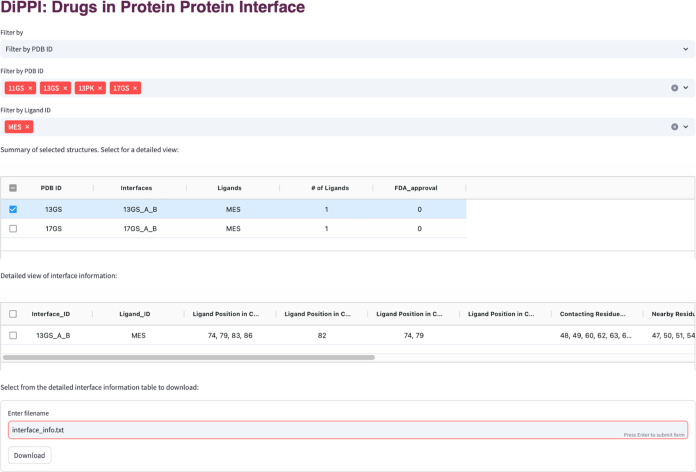
Example query for the “Query by Interface” page in DiPPI.

**Figure 5:**
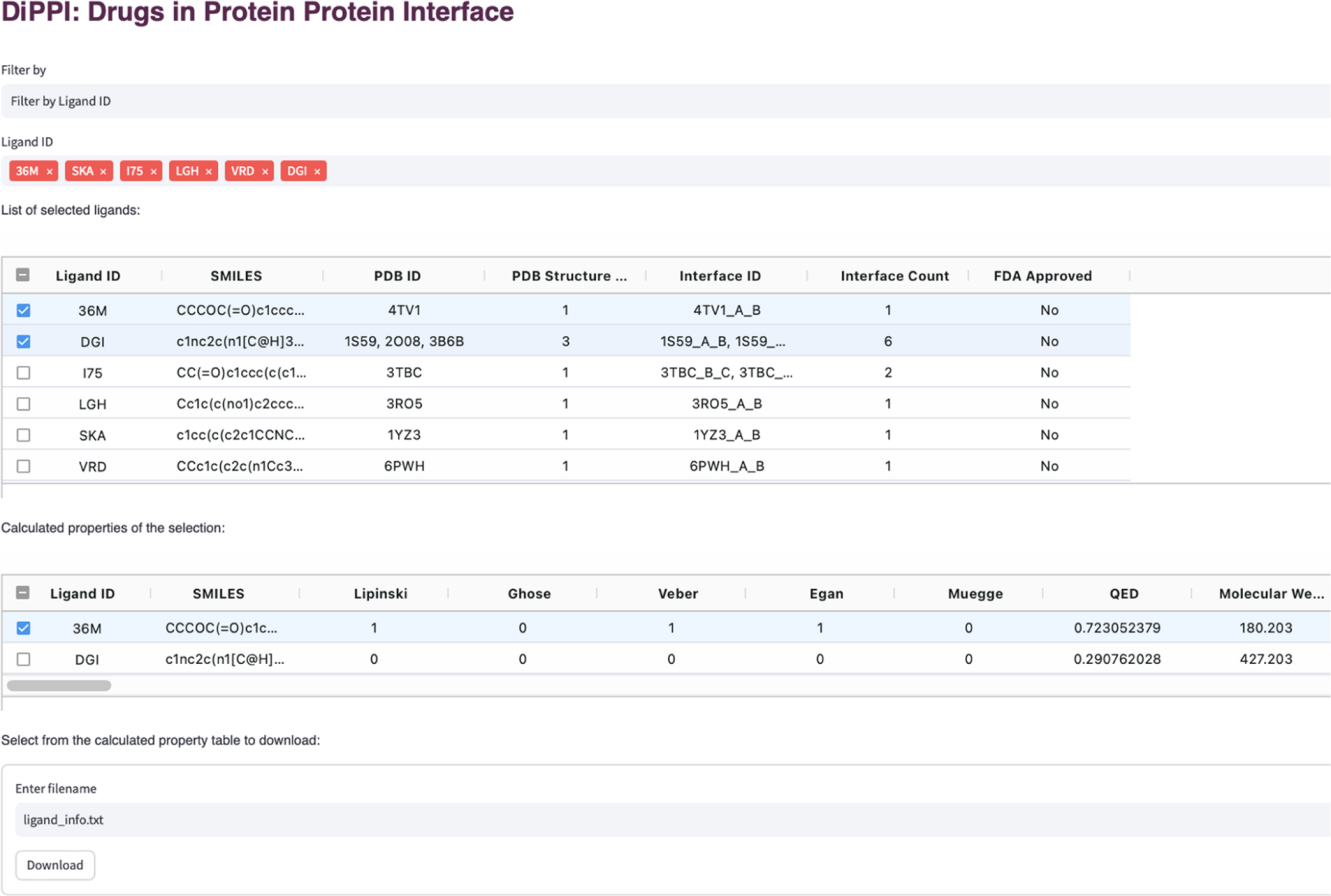
Example query for the “Query by Drug” page in DiPPI.

The DiPPI web site has two modules for the users to submit their queries. In the “Query by Interface” module, users can submit the PDB ID, UniProt ID, Protein Name, or Uniprot sequence of the protein of interest, together with an optional selection of the desired ligand to generate a downloadable table (Figure 4). The table contains information regarding interface residues, such as positions on the protein structure, position of the drug-like molecule on the interface, associated ligand ID, and hotpot residues if the drug-like molecule binds to them. In the “Query by Drug” module, users can search using the ligand ID or SMILES string of the ligand and retrieve a table with summary information about the drug and its associated interfaces (Figure 5). Upon clicking, the table shows detailed information about the selected ligands, including but not limited to calculated molecular descriptors and ligand cluster information. Tables on the pages are downloadable for further processing by the users.

### 3.6 Case Study

This web site provides the data for drug repurposing studies through the protein–protein interface and drug clusters. We tested the usability of our web site by performing an *in silico* novel drug identification for a protein interface, which can further be validated *in vitro*. For this, we selected a set of proteins with similar interfaces. There are 85 heterodimers in this set, 12 of which have a drug in their interfaces. We docked the drugs to other proteins in the set. This selection is based on the high number of unique FDA-approved drugs for different diseases that bind to the interfaces.

The unique drugs in this cluster, their usage, and some of their molecular descriptors are listed in Table S5. These drugs have a variety of uses, such as hypertension and hypertriglyceridemia treatment. The proteins in the selected structural cluster and the diseases to which they are related are given in Table S6. The proteins within the structural cluster are found to be related to numerous diseases, according to DisGeNET, including breast carcinoma, hypertensive disease, and liver-related diseases.

**Figure 6:**
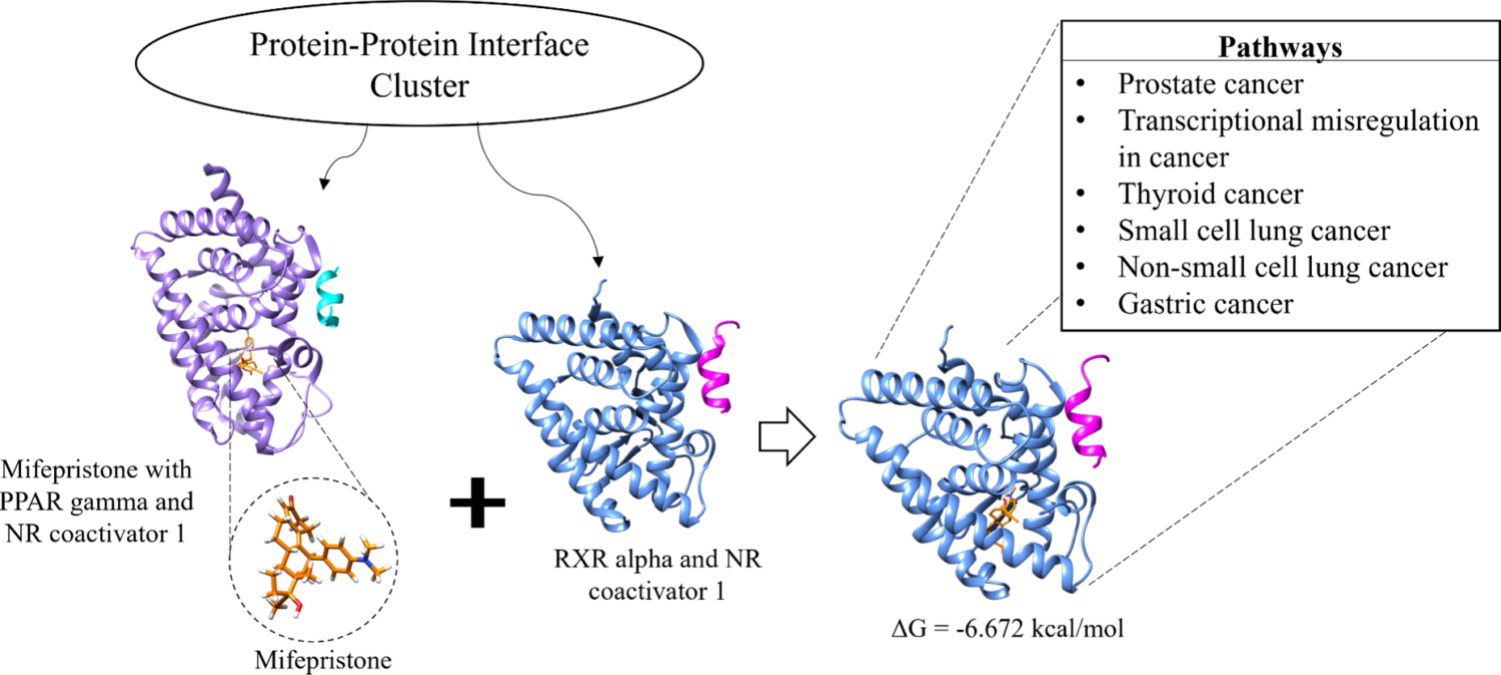
Mifepristone (orange) bound to the PPAR γ (medium purple)-nuclear receptor coactivator 1 (cyan) interface is docked to the RXR α (cornflower blue)-nuclear receptor coactivator 1 (pink) interface in the same protein–protein interface cluster found in DiPPI. As a result, mifepristone used for medical abortion is suggested to be repurposed for cancer treatment.

The seven unique FDA-approved drugs at the protein–protein interfaces are docked to other protein–protein interfaces in the same cluster and, hence, to the structurally similar protein–protein interfaces. There are 364 drug-interface combinations with this approach. Considering the two sides of the interface, 728 docking experiments were conducted. The mean binding energy of these dockings is −5.78 kcal/mol, whereas their median is −5.73 kcal/mol. The docking results, which had binding energy less than −6.5 kcal/mol, were considered to be promising. Among the docking results, we will discuss the case where mifepristone binds to the interface between retinoid X receptor α (RXRA) and nuclear receptor coactivator 1 (Figure 6) due to having supporting literature data, conserved protein–ligand interactions and structural evidence of the drug-binding region of the aligned structures. Mifepristone is a progesterone receptor antagonist used to terminate pregnancy up to 63 days gestation.^56^ In our results, mifepristone binds to RXRA with an energy of −6.672 kcal/mol. RXRA is a member of the nuclear receptor superfamily.^57^ Type 1 nuclear receptors activate transcriptional activities.^58^ The heterodimers that involve RXRA regulate transcriptional activities by binding to a coactivator.^59^ Deregulated transcription factors are frequently observed in human cancer,^60^ leading them to be considered as therapeutic targets, making RXRA and nuclear receptor coactivator 1 relevant targets.

RXRA is involved in pathways such as thyroid cancer, PI3K-Akt pathway, Hepatitis C, pathways in cancer, small cell lung cancer, nonsmall cell lung cancer, and gastric cancer in KEGG.^61^ In addition, RXRA is associated with prostatic neoplasms, according to DisGeNET. There are several studies for repurposing mifepristone as a cancer drug. A phase II study of mifepristone in castration-resistant prostate cancer (CRPC) suggested that mifepristone when combined with an agent that prevents the increase in adrenal androgens might benefit CRPC patients.^62,63^ Furthermore, another study reported the beneficial effects of mifepristone on murine testicular and prostate cancer.^64^ In addition, mifepristone is suggested to be inducing apoptosis for androgen-independent prostate cancer cells.^65^ Moreover, a combination of mifepristone and progesterone had an inhibitory effect on the growth of ovarian mesenchymal stem/stromal cells of females with the BRCA^1–/2–^ mutation having a higher risk of ovarian cancer. Hence, it is proposed for ovarian cancer prevention.^66^ In another study, inhibition of growth is observed in cancer cell lines from the nervous system, breast, prostate, ovary, and bone tissues with mifepristone by reducing the activity of Cdk2.^67^ The binding of mifepristone to RXRA might be another mechanism that reduces the rate of growth of cancer cells. AC50 of 0.99 μM is reported for binding of mifepristone to RXRA in T3DB.^68,69^ However, the bioactivity data on the potency of mifepristone on RXRA is reported to be inconclusive in ChEMBL.^70^ Additionally, it is reported that highly metastatic cancer cells exhibit decreased migration and invasion when exposed to mifepristone.^71^

**Figure 7:**
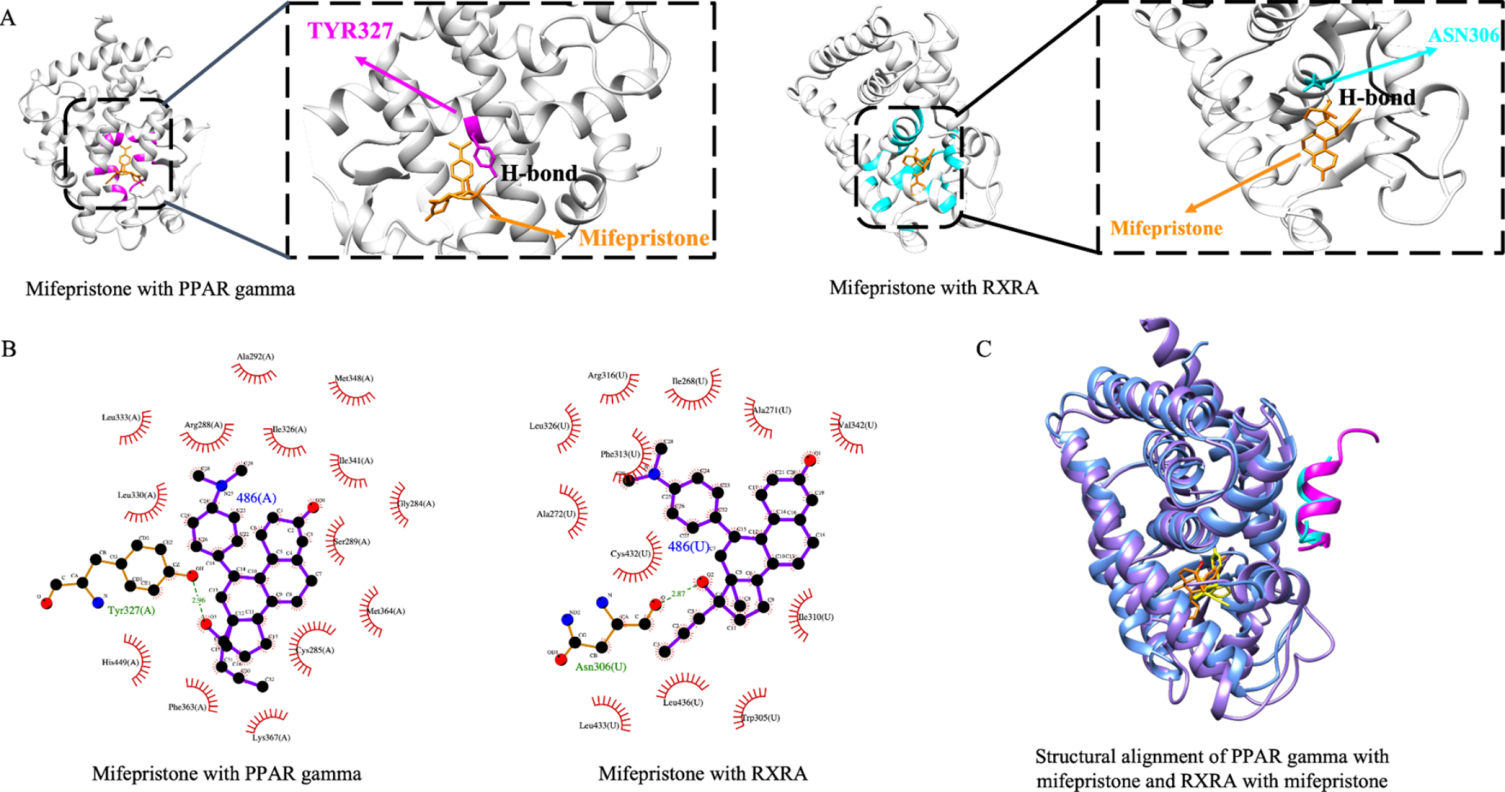
Similarity of PPAR γ with mifepristone and RXRA with mifepristone. Mifepristone is not directly bound to the interface since it is bound to a nearby residue of the interface. (A) Structural similarity of the mifepristone binding region in PPAR γ (PDB ID: 3QT0) and in RXRA (docked structure) is highlighted. (B) Protein–ligand interaction diagram for mifepristone with PPAR γ and mifepristone (ligand ID: 486) with RXRA.^72^ Hydrogen bonds are represented by green dashed lines. Hydrophobic contacts are represented by red spoked arcs. (C) Structural alignment of PPAR γ (medium purple) with mifepristone (yellow) and RXRA (cornflower blue) with mifepristone (orange) is shown. Both protein structures in cyan and pink are nuclear receptor coactivator 1.

When the binding region of mifepristone in its original target (PPAR γ) and in the proposed new target (RXRA) is analyzed, structural similarities are noticed. Figure 7A highlights the ligand contacting residues (i.e., the residues with a distance of less than 5 Å). In both cases, mifepristone is bound to a region with α helices. In addition, mifepristone binds to both proteins through an H-bond. The protein–ligand interaction diagram (Figure 7B) shows the H-bond between the mifepristone and PPAR γ and RXRA, as well as hydrophobic contacts. The structural alignments of PPAR γ and RXRA are shown in Figure 7C. Mifepristone binds to the same region in both cases. Moreover, Asn306 of RXRA forms an H-bond with mifepristone as Tyr327 of PPAR γ does.

Although the proposed mechanism might differ, there is various supporting evidence in the literature for repurposing mifepristone in cancer treatment that might be explained by protein–protein interface structural similarities at the molecular level.

## 4 Conclusions

In this study, we present DiPPI, a web site for the investigation of drug-like molecules in protein interfaces. Our web site provides users with a curated collection of drug-like molecules and their computed properties, along with the characterization of interfaces that contain a drug-like molecule in downloadable format. We investigated 534,203 predicted protein interfaces and 11,011 drug-like small molecules. Of all investigated molecules and interfaces, 335,648 interfaces are found to contain 2214 drug-like molecules. Our web site offers a guide to researchers who want to search for alternative targets and drugs in their drug repurposing studies by limiting the search space by allowing them to filter similar targets and drugs to their queries.

## Data Availability

The data sets generated and/or analyzed during the current study are available from the following GitHub repository: https://github.com/ku-cosbi/DiPPI/

## Supporting Information Available

Information on the selected molecular descriptors and their filtering based on the various criteria. Figure S1: Flowchart of the data curation process. Figure S2: Distribution of the drug-like molecules curated from the databases. Figure S3: Normalized distribution of Lipinski’s rules related to molecular descriptors of FDA-approved drugs. Figure S4: Normalized distribution of Lipinski’s rules related to molecular descriptors of drugs in interfaces. Table S1: Ligand cluster characterization for ECFP4 and pharmacophore fingerprints. Table S2: Description of the data set. Table S3: Top 10 most occurring KEGG pathways in FDA-approved drug-bound interfaces data set. Table S4: Top 10 most occurring Pfam families in FDA-approved drug-bound interfaces data set. Table S5: Drugs docked in the case study, their usage, and the following molecular descriptors: molecular weights (MWt), the number of hydrogen acceptors (HA), the number of hydrogen donors, and the number of rotatable bonds. Table S6: Proteins that are in the structural cluster (with the cluster representative 5AZT_A_C) and the diseases they are the most related to. Only the diseases with DisGeNET scores higher than or equal to 0.5 are given (PDF)
