# Supplementary material for: DiPPI: A Curated Data Set for Drug-like Molecules in Protein–Protein Interfaces
Source: J Chem Inf Model. 2024 Jun 22;64(13):5041–51. doi: 10.1021/acs.jcim.3c01905 (PMC11577314; doi:10.1021/acs.jcim.3c01905)
Supplement: Supplementary file 1 — ci3c01905_si_001.pdf [file ci3c01905_si_001.pdf]

Supporting Information

DiPPI: A curated dataset for drug-like molecules in protein-protein interfaces

Fatma Cankara[1], Simge Senyuz[1*], Ahenk Zeynep Sayin[2], Attila Gursoy[3] and Ozlem Keskin[2#]*

1. Graduate School of Sciences and Engineering, Koç University, İstanbul, 34450, Turkey
2. Department of Chemical and Biological Engineering, Koç University, İstanbul, 34450, Turkey
3. Department of Computer Engineering, Koç University, İstanbul, 34450, Turkey

* Equal contribution
# Corresponding author

Defining and Clustering the Protein-Protein Interfaces

Protein-protein interfaces are identified by extracting all PDB structures containing more than two protein chains. If the total SASA of the monomer is larger than that of the dimer by at least 1Å, then the dimer is considered to have an interface. Protein-protein interfaces were defined as a combination of residues that either directly contact or are in close proximity to each other across the dimer interface. Contacting residues are characterized by atoms whose distances from each other do not exceed the sum of their respective van der Waals (vdW) radii plus a threshold distance of 0.5Å. The vdW radii utilized for computing inter-atomic distances are extracted from the CHARMM (Chemistry at Harvard Macromolecular Mechanics)[1] force field definitions governing vdW parameters. Furthermore, any residue with a Cα atom within a 6Å range from an atom of a contacting residue is classified as a nearby residue. For a structure to be defined as an interface, there must be at least 5 amino acids present in both chains.

Different conformations of interfaces occurring between a protein dimer structure are identified by first using MMSeqs2[2] with a 95% similarity threshold to cluster protein chains based on their

sequences. Following the sequence clustering, interfaces that occur as a result of the interaction of monomers that are members of the same cluster were identified. In other words, if the first chain of protein A and the first chain of protein B are in the same cluster, and the second chain of protein A and the second chain of protein B are in the same cluster, then these interfaces are considered to be formed between two homologous dimers, irrespective of their interaction sites. After grouping interfaces that belong to the same dimer sequences, the interface regions are structurally compared within groups using iAlign[3] to determine the different conformations of interfaces or different interfaces within a dimer group. Structurally similar interfaces within the same sequence clusters are further clustered using an agglomerative hierarchical clustering algorithm based on the IS-score values, which serve as the similarity criterion. A similarity threshold of 0.311 of IS-score, equivalent to a p-value of 10^{-5}, was employed. Following the sequence and structural similarity clustering, interface representatives were selected within their clusters based on the total similarity to other interfaces in the same cluster.

Supporting Information on Dataset Characterization

The molecular descriptors are filtered based on the following criteria:

1. Lipinski's Rule of Five[4]:
    a. Lipophilicity (logP) < 5
    b. Molecular weight (MW) < 500
    c. The number of hydrogen bond donors (HBD) < 5
    d. The number of hydrogen bond acceptors (HBA) < 10
2. Ghose's criteria[5]:
    a. $160 \leq$ Molecular weight (MW) ≤ 480
    b. $-0.4 \leq$ Lipophilicity (logP) ≤ 5.6
    c. $40 \leq$ Molar refractivity (MR) ≤ 130
    d. $20 \leq$ The number of atoms ≤ 70
3. Veber's criteria[6]:
    a. The number of rotatable bonds ≤ 10
    b. Polar surface area (PSA) ≤ 140
4. Egan's criteria[7]:
    a. Lipophilicity (logP) $\leq 5,88$
    b. Polar surface area (PSA) $\leq 131,6$
5. Muegge's criteria[8]:
    a. $200 \leq$ Molecular weight (MW) ≤ 600
    b. $-2 \leq$ Lipophilicity (logP) ≤ 5
    c. Polar surface area (PSA) ≤ 150

d. The number of rings ≤ 7
e. The number of carbons > 4
f. The number of heteroatoms > 1
g. The number of rotatable bonds (ROTB) ≤ 15
h. The number of hydrogen bond acceptors (HBA) ≤ 10
i. The number of hydrogen bond donors (HBD) ≤ 5

6. Quantitative estimate of drug-likeness (QED)[9]:
   a. QED score is calculated with Python's RDKit module. QED score uses the following parameters: octanol-water partition coefficient (ALOGP), molecular weight (MW), the number of hydrogen bond acceptors (HBA), the number of hydrogen bond donors (HBD), polar surface area (PSA), rotatable bond count (ROTB), aromatic ring count (AROM), the presence of unwanted chemical functionalities / structural alerts (ALERTS)

Supporting Figures

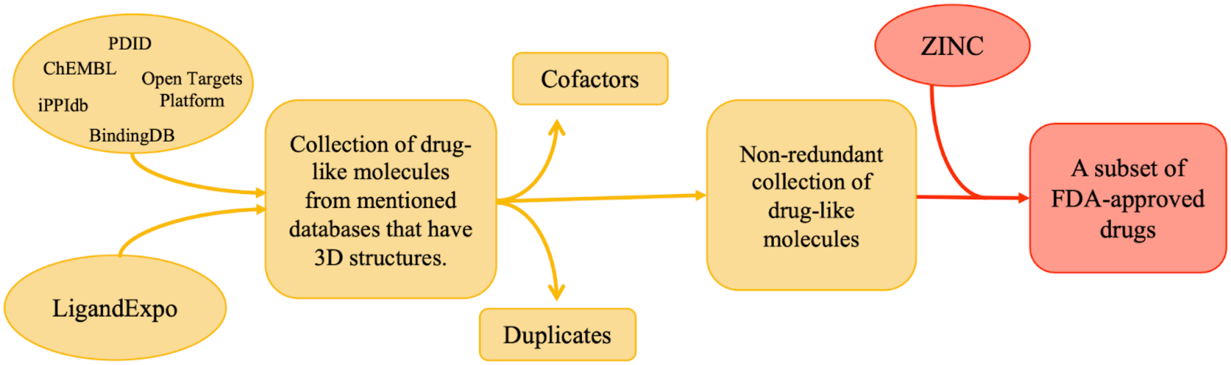

Figure S1. Flowchart depicting the data acquisition process for drug-like molecules.

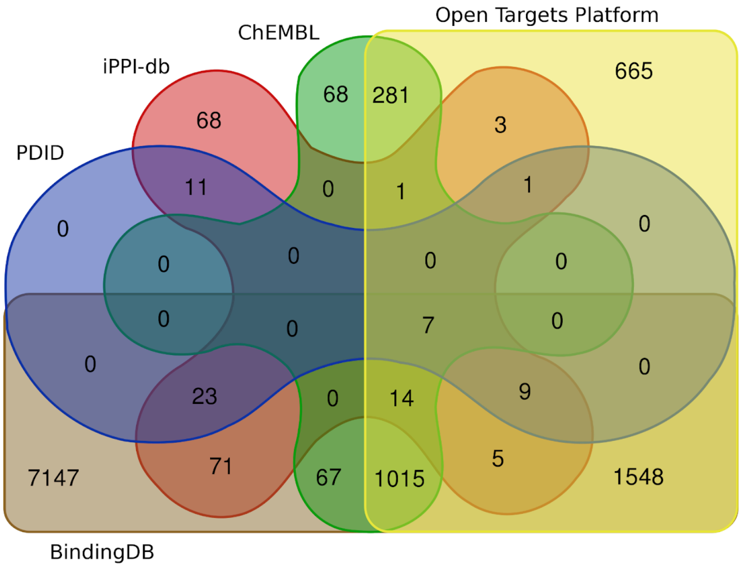

Figure S2. The distribution of drug-like molecules taken from ChEMBL, iPPIdb, PDID, Open Targets Platform and BindingDB. Venn diagram shows the number of shared and unique ligands obtained from each source database. Red: iPPI-db, Green: CHEMBL, Yellow: Open Targets Platform, Blue: PDID, Brown: BindingDB.

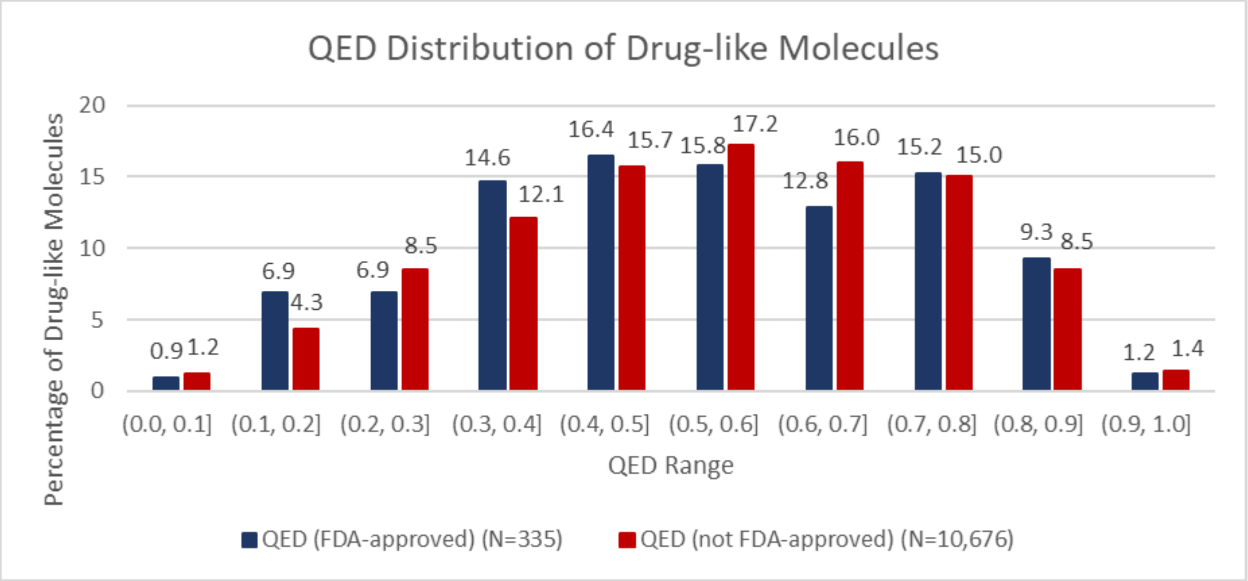

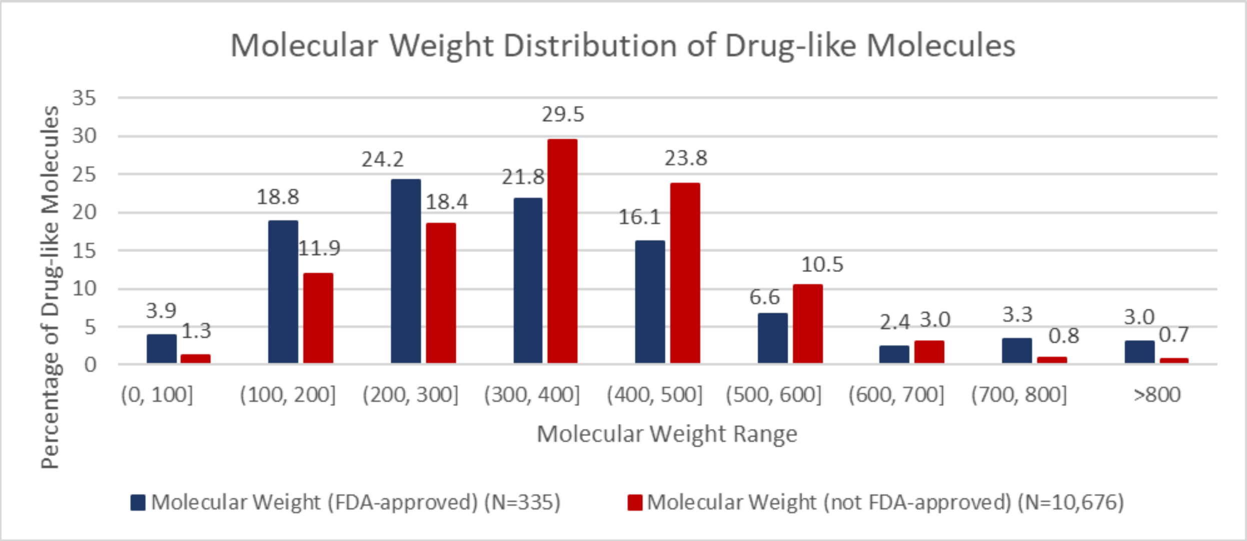
Molecular Weight Distribution of Drug-like Molecules
Percentage of Drug-like Molecules
35
30
25
20
15
10
5
0
3.9
1.3
18.8
11.9
24.2
18.4
21.8
29.5
16.1
23.8
6.6
10.5
2.4
3.0
3.3
0.8
3.0
0.7
(0, 100]
(100, 200]
(200, 300]
(300, 400]
(400, 500]
(500, 600]
(600, 700]
(700, 800]
>800
Molecular Weight Range
Molecular Weight (FDA-approved) (N=335)
Molecular Weight (not FDA-approved) (N=10,676)

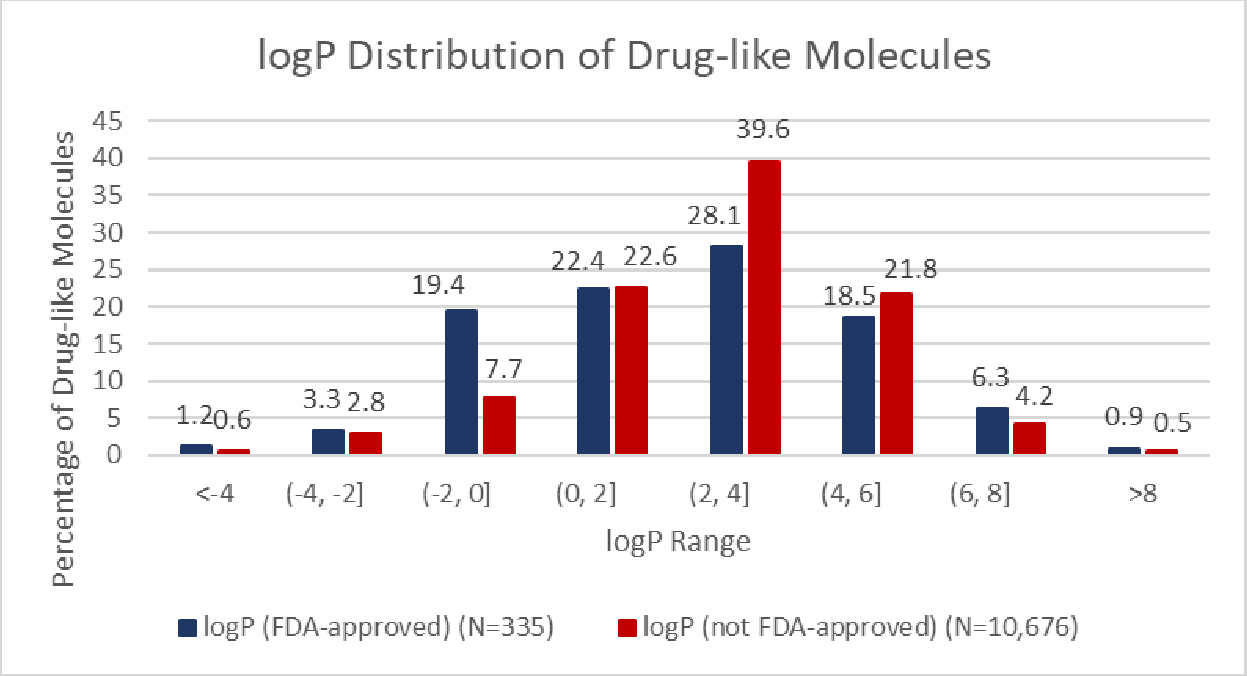
logP Distribution of Drug-like Molecules
Percentage of Drug-like Molecules
45
40
35
30
25
20
15
10
5
0
1.2
0.6
3.3
2.8
19.4
7.7
22.4
22.6
28.1
39.6
18.5
21.8
6.3
4.2
0.9
0.5
<-4
(-4, -2]
(-2, 0]
(0, 2]
(2, 4]
(4, 6]
(6, 8]
>8
logP Range
logP (FDA-approved) (N=335)
logP (not FDA-approved) (N=10,676)

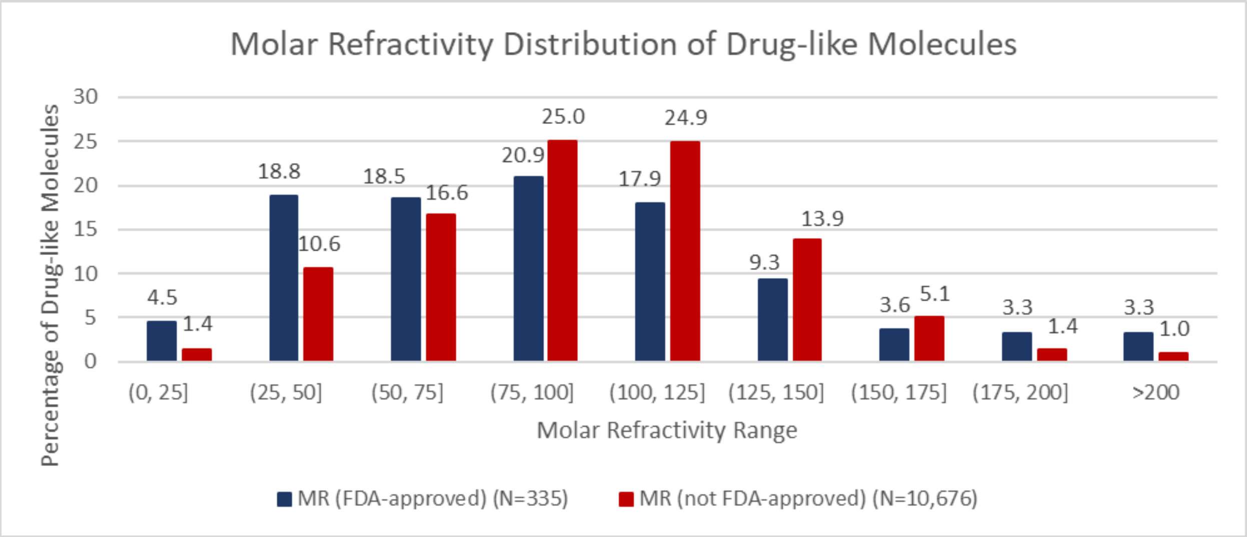
Molar Refractivity Distribution of Drug-like Molecules
Percentage of Drug-like Molecules
30
25
20
15
10
5
0
4.5
1.4
18.8
10.6
18.5
16.6
20.9
25.0
17.9
24.9
9.3
13.9
3.6
5.1
3.3
1.4
3.3
1.0
(0, 25]
(25, 50]
(50, 75]
(75, 100]
(100, 125]
(125, 150]
(150, 175]
(175, 200]
>200
Molar Refractivity Range
MR (FDA-approved) (N=335)
MR (not FDA-approved) (N=10,676)

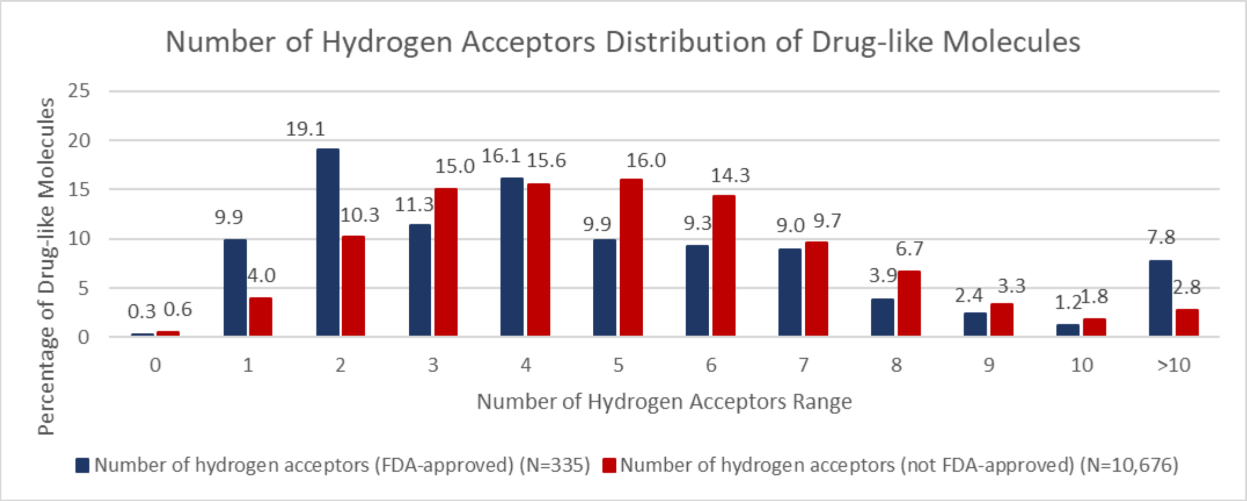
Number of Hydrogen Acceptors Distribution of Drug-like Molecules
Percentage of Drug-like Molecules
25
20
15
10
5
0
0.3
0.6
9.9
4.0
19.1
10.3
11.3
15.0
16.1
15.6
9.9
16.0
9.3
14.3
9.0
9.7
3.9
6.7
2.4
3.3
1.2
1.8
7.8
2.8
0
1
2
3
4
5
6
7
8
9
10
>10
Number of Hydrogen Acceptors Range
Number of hydrogen acceptors (FDA-approved) (N=335)
Number of hydrogen acceptors (not FDA-approved) (N=10,676)

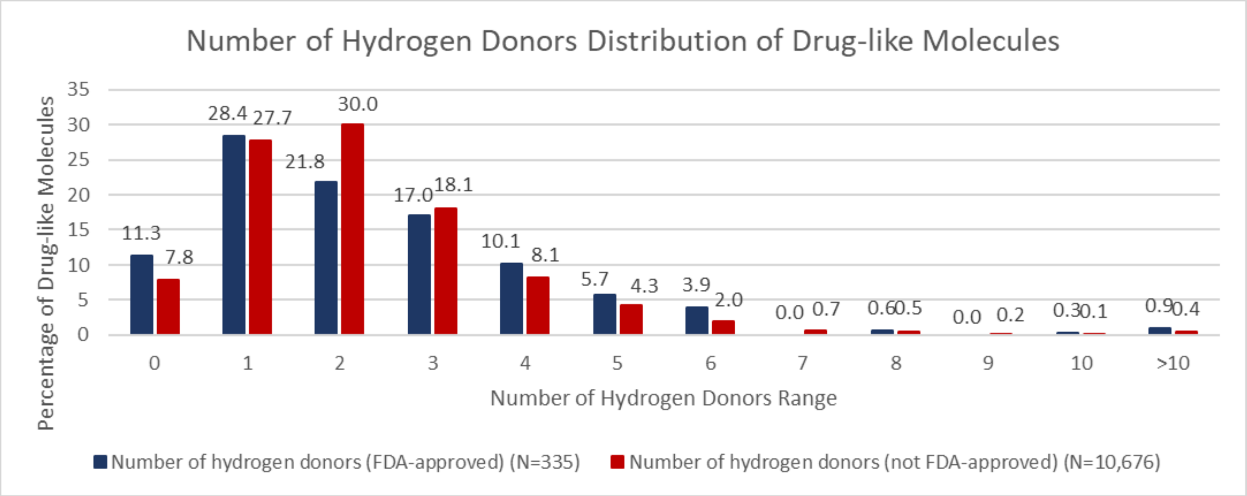
Number of Hydrogen Donors Distribution of Drug-like Molecules
Percentage of Drug-like Molecules
35
30
25
20
15
10
5
0
11.3
7.8
28.4
27.7
21.8
30.0
17.0
18.1
10.1
8.1
5.7
4.3
3.9
2.0
0.0
0.7
0.6
0.5
0.0
0.2
0.3
0.1
0.9
0.4
0
1
2
3
4
5
6
7
8
9
10
>10
Number of Hydrogen Donors Range
Number of hydrogen donors (FDA-approved) (N=335)
Number of hydrogen donors (not FDA-approved) (N=10,676)

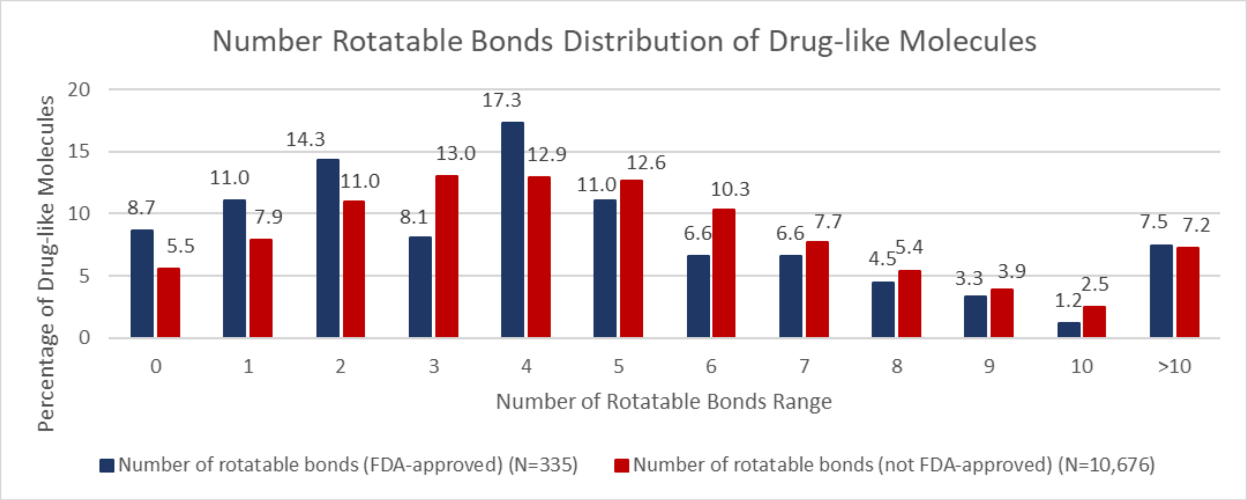

Figure S3. Normalized distribution of Lipinski's rules related to molecular descriptors of FDA-approved drugs (N=335) and not FDA-approved drugs (N=10,676).

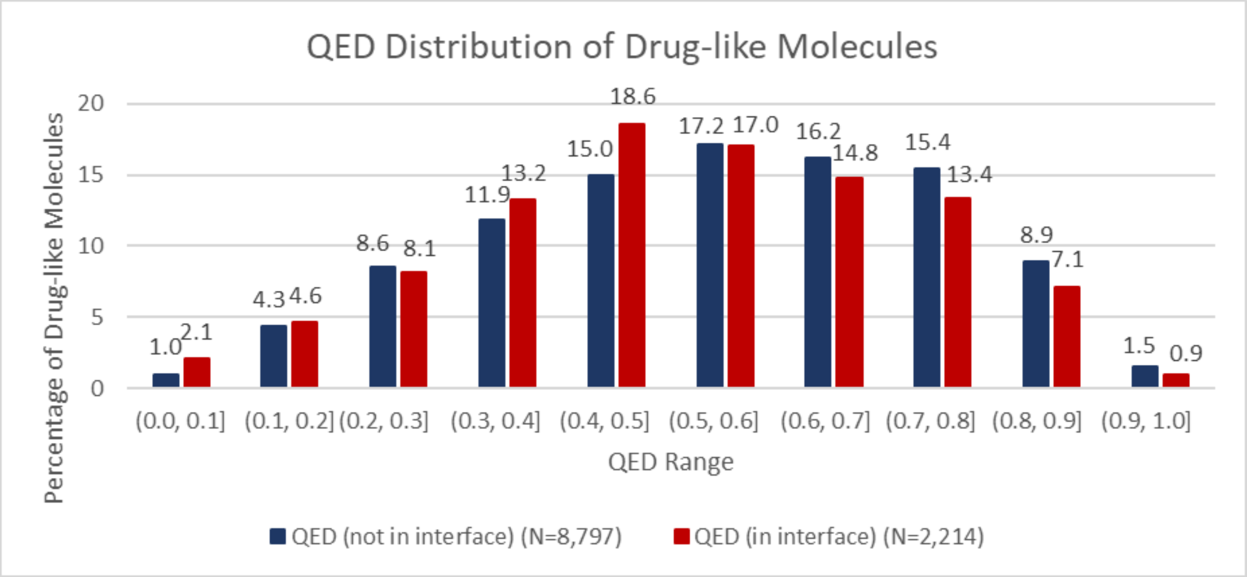

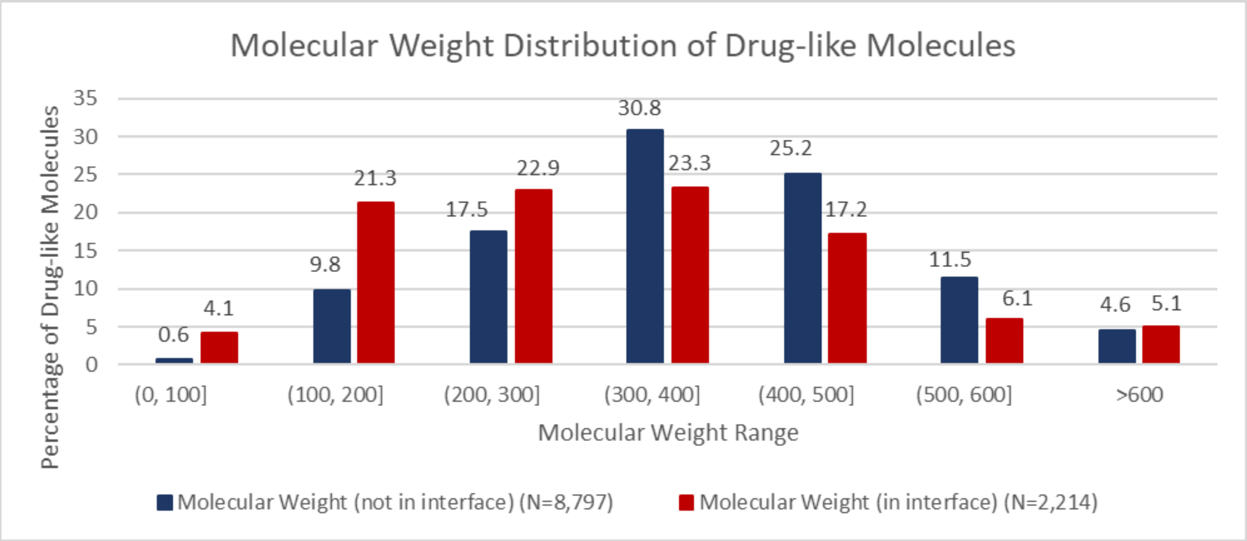
Molecular Weight Distribution of Drug-like Molecules
Percentage of Drug-like Molecules
35
30
25
20
15
10
5
0
0.6
4.1
9.8
21.3
17.5
22.9
30.8
23.3
25.2
17.2
11.5
6.1
4.6
5.1
(0, 100]
(100, 200]
(200, 300]
(300, 400]
(400, 500]
(500, 600]
>600
Molecular Weight Range
Molecular Weight (not in interface) (N=8,797)
Molecular Weight (in interface) (N=2,214)

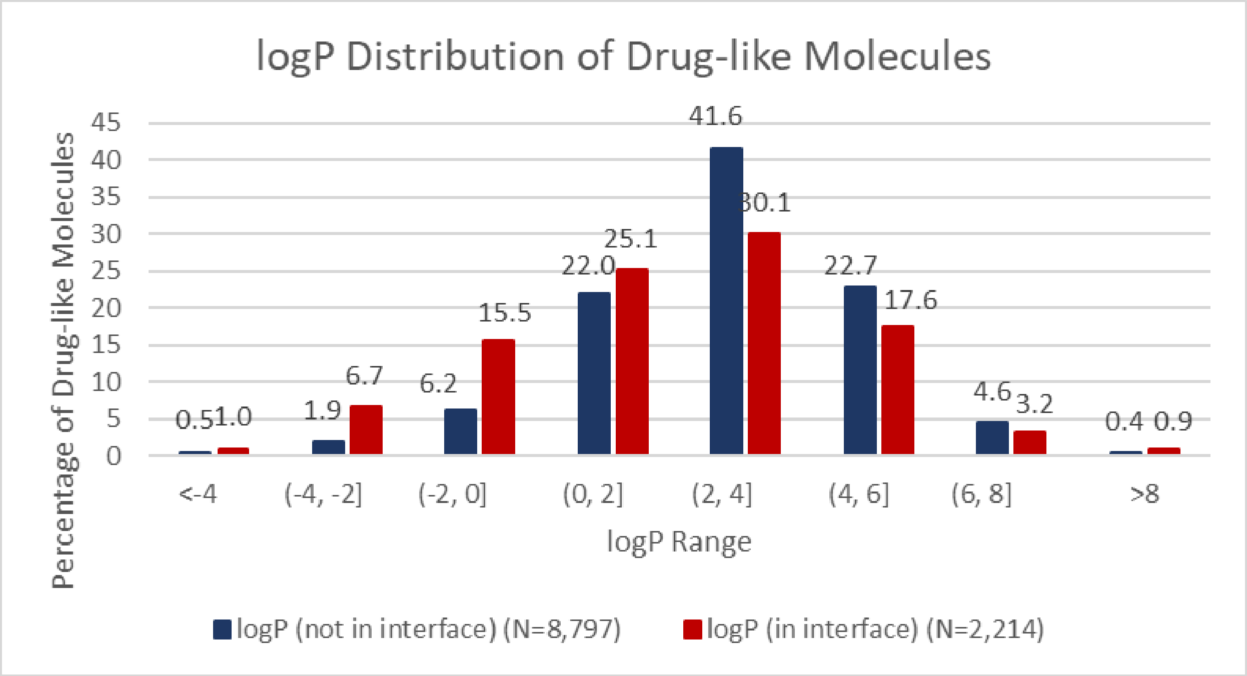
logP Distribution of Drug-like Molecules
Percentage of Drug-like Molecules
45
40
35
30
25
20
15
10
5
0
0.5
1.0
1.9
6.7
6.2
15.5
22.0
25.1
41.6
30.1
22.7
17.6
4.6
3.2
0.4
0.9
<-4
(-4, -2]
(-2, 0]
(0, 2]
(2, 4]
(4, 6]
(6, 8]
>8
logP Range
logP (not in interface) (N=8,797)
logP (in interface) (N=2,214)

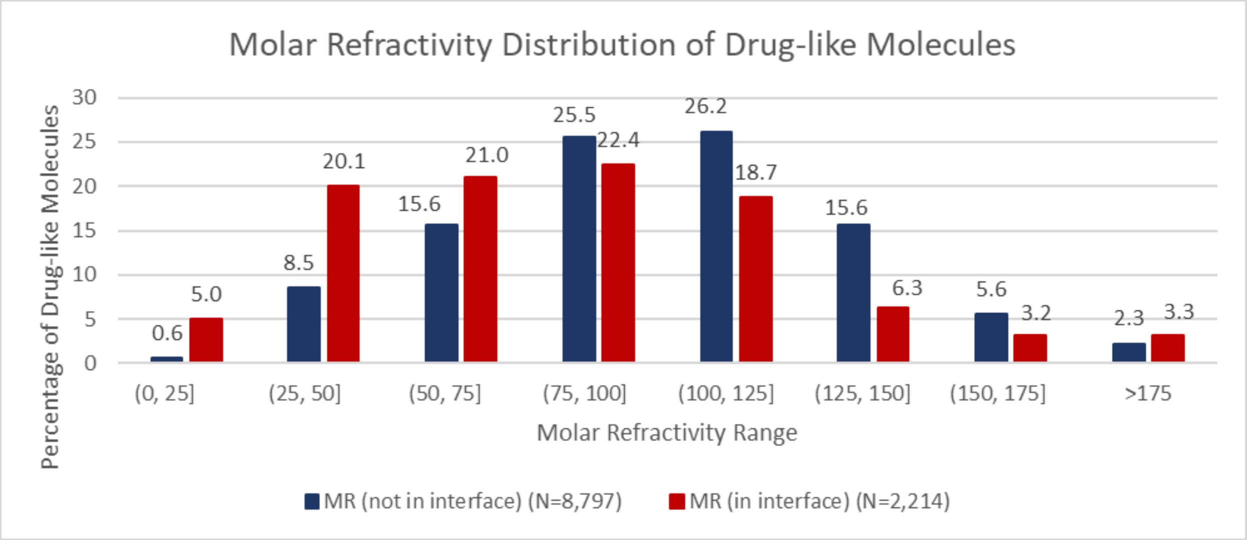
Molar Refractivity Distribution of Drug-like Molecules
Percentage of Drug-like Molecules
30
25
20
15
10
5
0
0.6
5.0
8.5
20.1
15.6
21.0
25.5
22.4
26.2
18.7
15.6
6.3
5.6
3.2
2.3
3.3
(0, 25]
(25, 50]
(50, 75]
(75, 100]
(100, 125]
(125, 150]
(150, 175]
>175
Molar Refractivity Range
MR (not in interface) (N=8,797)
MR (in interface) (N=2,214)

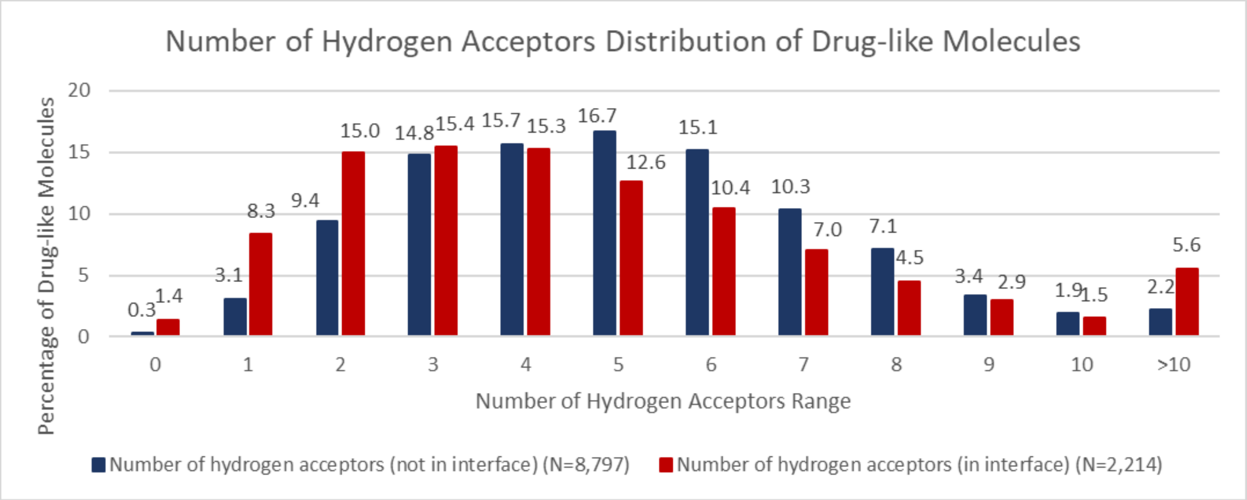
Number of Hydrogen Acceptors Distribution of Drug-like Molecules
Percentage of Drug-like Molecules
20
15
10
5
0
0.3
1.4
3.1
8.3
9.4
15.0
14.8
15.4
15.7
15.3
16.7
12.6
15.1
10.4
10.3
7.0
7.1
4.5
3.4
2.9
1.9
1.5
2.2
5.6
0
1
2
3
4
5
6
7
8
9
10
>10
Number of Hydrogen Acceptors Range
Number of hydrogen acceptors (not in interface) (N=8,797)
Number of hydrogen acceptors (in interface) (N=2,214)

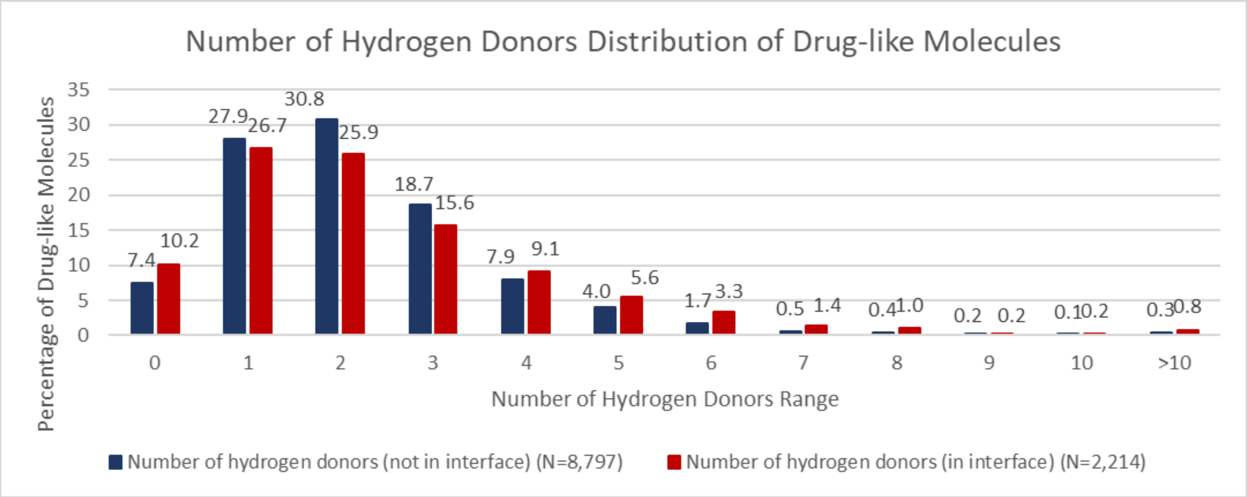
Number of Hydrogen Donors Distribution of Drug-like Molecules
Percentage of Drug-like Molecules
35
30
25
20
15
10
5
0
7.4
10.2
27.9
26.7
30.8
25.9
18.7
15.6
7.9
9.1
4.0
5.6
1.7
3.3
0.5
1.4
0.4
1.0
0.2
0.2
0.1
0.2
0.3
0.8
0
1
2
3
4
5
6
7
8
9
10
>10
Number of Hydrogen Donors Range
Number of hydrogen donors (not in interface) (N=8,797)
Number of hydrogen donors (in interface) (N=2,214)

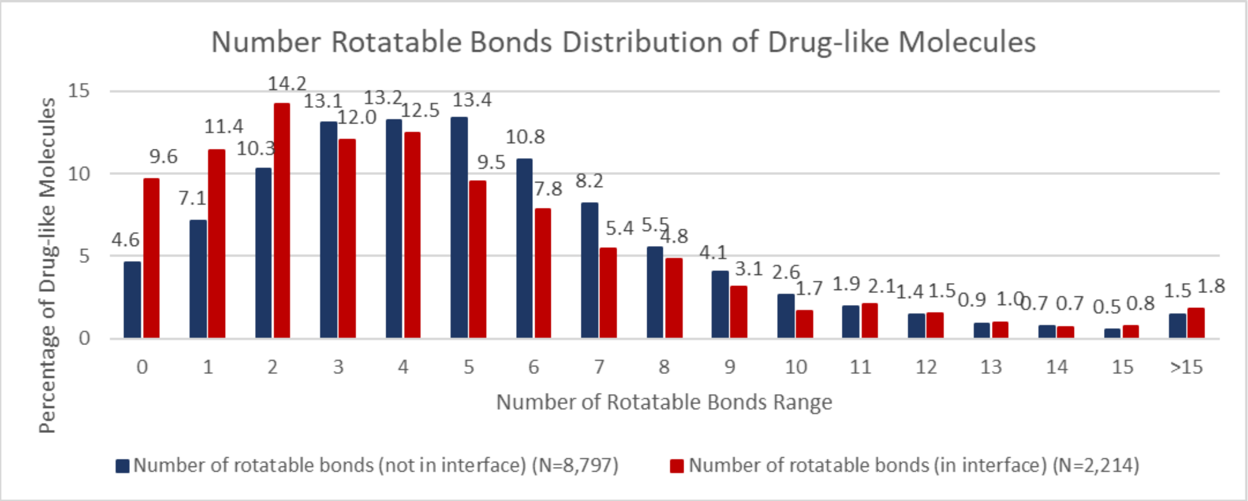

Figure S4. Normalized distribution of Lipinski's rules related to molecular descriptors of drugs in interfaces (N=2,214) and not in interfaces (N=8,797).

Supporting Tables

Table S1. Ligand cluster characterization for ECFP4 and pharmacophore fingerprints

	ECFP4	Pharmacophore
Selected threshold value	0.6	0.5
Total cluster number	6084	2799
Clusters with only one molecule	4270	1473
Clusters with more than 5 molecules	242	383
Clusters with more than 10 molecules	53	162
Clusters with more than 25 molecules	10	51
Clusters with more than 50 molecules	1	23
Molecule count in the most crowded cluster	93	303
Similarity between two random molecules within a cluster	0.66	0.65

Similarity between two random molecules in two random clusters	0.07	0.16

Table S2. The description of the dataset

Number of investigated proteins	98,632
Number of investigated interfaces	534,203
Number of interfaces belonging to proteins with bound drugs (any region)	335,648
Number of interfaces to which at least one drug-like molecule binds to	53,452
Number of interfaces to which at least one FDA-drug binds to	19,960
Number of investigated drug-like small molecules	11,011
Number of eliminated small molecules	402
Number of investigated FDA-approved drugs	1,615
Number of drug-like small molecules that bind to at least one interface residue	2,214
Number of FDA-approved drugs that bind to at least one interface residue	335

Table S3. Top 10 most occurring KEGG pathways in FDA-approved drug-bound interfaces dataset

**KEGG Pathway Name**	**KEGG Pathway ID**	**Number of Occurences**
Metabolic pathways	01100	9342
Biosynthesis of secondary metabolites	01110	2524

Alzheimer disease	05010	1888
Biosynthesis of cofactors	01240	1770
Microbial metabolism in diverse environments	01120	1543
Diabetic cardiomyopathy	05415	1456
Pathways of neurodegeneration - multiple diseases	05022	1446
Non-alcoholic fatty liver disease	04932	1324
Type I diabetes mellitus	04940	1310
Human T-cell leukemia virus 1 infection	05166	1275

Table S4. Top 10 most occurring Pfam families in FDA-approved drug-bound interfaces dataset

**Pfam Family Name**	**Pfam ID**	**Number of Occurrences**
Immunoglobulin C1-set domain	PF07654	1794
Reverse transcriptase connection domain	PF06815	924
Reverse transcriptase thumb domain	PF06817	924
Reverse transcriptase (RNA-dependent DNA polymerase)	PF00078	924
Glycosyl hydrolases family 2, TIM barrel domain	PF02836	818
Glycosyl hydrolases family 2	PF00703	816
Glycosyl hydrolases family 2, sugar binding domain	PF02837	816
Beta galactosidase small chain	PF02929	720
Beta-galactosidase, domain 4	PF16353	720

Neurotransmitter-gated ion-channel ligand binding domain	PF02931	660

Table S5. The drugs docked in the case study, their usage, and the following molecular descriptors: molecular weights (MWt), the number of hydrogen acceptors (HA), the number of hydrogen donors, and the number of rotatable bonds.

**Drug Name**	**Ligand ID**	**Usage**	**MWt**	**logP**	**HA**	**HD**	**ROTB**
Bexarotene	9RA	Used for the treatment of the skin manifestations of CTCL	348.49	6.10	1	1	3
Alitretinoin	9CR	Used for the treatment of the lesions in patients with AIDS-related Kaposi's sarcoma	300.44	5.60	1	1	5
Mifepristone	486	Used to terminate intrauterine pregnancy	429.60	5.41	3	1	2
Docosahexaenoic acid	HXA	An omega-3 fatty acid	328.50	6.55	1	1	14
Fenofibric acid	F5A	Supplementary to the treatment of hypertriglyceridemia and high cholesterol	318.75	3.81	3	1	5
Telmisartan	TLS	Used for the treatment of hypertension	514.63	7.26	5	1	7
Eicosapentaenoic Acid	EPA	Related to Icosapent which is used in the treatment of hyperglyceridemia	302.46	5.99	1	1	13

Table S6. The proteins that are in the structural cluster (with the cluster representative 5AZT_A_C) and the diseases they are the most related to. Only the diseases with DisGeNET scores higher than or equal to 0.5 are given.

Protein	Protein Name	Uniprot ID	Disease	Drug Bound Interfaces within the Cluster
NCOA1	Nuclear receptor coactivator 1	Q15788	Breast carcinoma	3QT0_A_C, 1FM9_A_B, 1FM6_A_B, 1K74_A_B, 7BQ4_A_B, 7BQ0_A_B, 3VN2_A_C
NCOA2	Nuclear receptor coactivator 2	Q15596	No results found with high scores	3OAP_A_B, 4NQA_A_C, 4K6I_A_B, 1MV9_A_B
Nr0b2	Nuclear receptor subfamily 0 group B member 2	P97947	No results found	This protein is in the cluster but none of the drugs are bound to its interfaces
RXRA	Retinoic acid receptor RXR-alpha	P19793, P28700	Prostatic neoplasms	1FM9_A_B, 1FM6_A_B, 1K74_A_B, 1XLS_A_I, 3OAP_A_B, 4NQA_A_C, 4K6I_A_B, 1MV9_A_B, 1XDK_A_C
RXRB	Retinoic acid receptor RXR-beta	P28702	No results found with high scores	Protein is in the cluster but none of the drugs are bound to its interfaces

Med1	Mediator of RNA polymerase II transcription subunit 1	Q925J9	No results found	1XDK_A_C
PPARA	Peroxisome proliferator-activated receptor alpha	Q07869	Fatty liver, liver neoplasms, hypertensive disease, malignant neoplasm of liver, reperfusion injury	7BQ4_A_B, 7BQ0_A_B
PPARG	Peroxisome proliferator-activated receptor gamma	P37231	Obesity, Familial Partial Lipodystrophy (Type 3), Diabetes Mellitus (Non-Insulin-Dependent), Hypertensive disease, Malignant tumor of colon, Diabetic Nephropathy, Familial partial lipodystrophy, Inflammation, Diabetes Mellitus, Atherosclerosis, Colorectal Carcinoma, Acute kidney injury, Acute Lung Injury, Glomerulonephritis, Familial Partial Lipodystrophy Type 1), Carotid Intimal Medial Thickness 1	3QT0_A_C, 3VN2_A_C

References

(1) Brooks, B. R.; Brooks, C. L., 3rd; Mackerell, A. D., Jr; Nilsson, L.; Petrella, R. J.; Roux, B.; Won, Y.; Archontis, G.; Bartels, C.; Boresch, S.; Caflisch, A.; Caves, L.; Cui, Q.; Dinner, A. R.; Feig, M.; Fischer, S.; Gao, J.; Hodoscek, M.; Im, W.; Kuczera, K.; Lazaridis, T.; Ma, J.; Ovchinnikov, V.; Paci, E.; Pastor, R. W.; Post, C. B.; Pu, J. Z.; Schaefer, M.; Tidor, B.; Venable, R. M.; Woodcock, H. L.; Wu, X.; Yang, W.; York, D. M.; Karplus, M. CHARMM: The Biomolecular Simulation Program. *J. Comput. Chem.* **2009**, *30* (10), 1545–1614.

(2) Steinegger, M.; Söding, J. MMseqs2 Enables Sensitive Protein Sequence Searching for the Analysis of Massive Data Sets. *Nat. Biotechnol.* **2017**, *35* (11), 1026–1028.

(3) Gao, M.; Skolnick, J. iAlign: A Method for the Structural Comparison of Protein-Protein Interfaces. *Bioinformatics* **2010**, *26* (18), 2259–2265.

(4) Lipinski, C. A.; Lombardo, F.; Dominy, B. W.; Feeney, P. J. Experimental and Computational Approaches to Estimate Solubility and Permeability in Drug Discovery and Development Settings. *Adv. Drug Deliv. Rev.* **2001**, *46* (1-3), 3–26.

(5) Ghose, A. K.; Viswanadhan, V. N.; Wendoloski, J. J. A Knowledge-Based Approach in Designing Combinatorial or Medicinal Chemistry Libraries for Drug Discovery. 1. A Qualitative and Quantitative Characterization of Known Drug Databases. *J. Comb. Chem.* **1999**, *1* (1), 55–68.

(6) Veber, D. F.; Johnson, S. R.; Cheng, H.-Y.; Smith, B. R.; Ward, K. W.; Kopple, K. D. Molecular Properties That Influence the Oral Bioavailability of Drug Candidates. *J. Med. Chem.* **2002**, *45* (12), 2615–2623.

(7) Egan, W. J.; Merz, K. M., Jr; Baldwin, J. J. Prediction of Drug Absorption Using Multivariate Statistics. *J. Med. Chem.* **2000**, *43* (21), 3867–3877.

(8) Muegge, I. Pharmacophore Features of Potential Drugs. *Chemistry* **2002**, *8* (9), 1976–1981.

(9) Bickerton, G. R.; Paolini, G. V.; Besnard, J.; Muresan, S.; Hopkins, A. L. Quantifying the Chemical Beauty of Drugs. *Nat. Chem.* **2012**, *4* (2), 90–98.
